# A flow reactor setup for photochemistry of biphasic gas/liquid reactions

**DOI:** 10.3762/bjoc.12.170

**Published:** 2016-08-11

**Authors:** Josef Schachtner, Patrick Bayer, Axel Jacobi von Wangelin

**Affiliations:** 1 Institute of Organic Chemistry, University of Regensburg, Universitaetsstr. 31, 93040 Regensburg, Germany

**Keywords:** biphasic reactions, flow chemistry, gas phase, microreactor, oxygen, photochemistry

## Abstract

A home-built microreactor system for light-mediated biphasic gas/liquid reactions was assembled from simple commercial components. This paper describes in full detail the nature and function of the required building elements, the assembly of parts, and the tuning and interdependencies of the most important reactor and reaction parameters. Unlike many commercial thin-film and microchannel reactors, the described set-up operates residence times of up to 30 min which cover the typical rates of many organic reactions. The tubular microreactor was successfully applied to the photooxygenation of hydrocarbons (Schenck ene reaction). Major emphasis was laid on the realization of a constant and highly reproducible gas/liquid slug flow and the effective illumination by an appropriate light source. The optimized set of conditions enabled the shortening of reaction times by more than 99% with equal chemoselectivities. The modular home-made flow reactor can serve as a prototype model for the continuous operation of various other reactions at light/liquid/gas interfaces in student, research, and industrial laboratories.

## Introduction

The recent developments of microreactor technologies have significantly impacted the art of organic synthesis and manufacture [1–7]. The efficiency of chemical reactions can be greatly enhanced over common batch processes and new approaches to the optimization of established reaction protocols and the execution of hitherto unfeasible processes can be enabled due to the inherent properties of micro/flow reactors: high mass-transfer rates [8], spatial separation of reagent addition and mixing, high reagent dispersion, high energy efficiency, improved irradiation [9–11], ease of upscaling, low hazard potential and multidimensional parameter control [7,9,11–12]. Over the past decade, various reactor types and technical specifications have been developed to address the intricate challenges of many chemical reactions, including the handling of hazardous [13–14] or explosive [15–16] reagents, advanced concentration and temperature gradients [17], multiphasic reactions including solid-phase protocols [18], addition of gaseous reagents [19], high-pressure conditions [20], cascade conversions without intermediate work-up operations [21], as well as thin film, falling film [22], micro-channel [23], and tube-in-tube reactors [24–25] for reactions between gaseous and liquid components. The high energy efficiency, low hazard potential, and precise control of reaction parameters have also prompted several adoptions of microflow techniques in technical manufactures of fine chemicals, polymers [26], and pharmaceutical intermediates [27–30].

**Figure 1:**
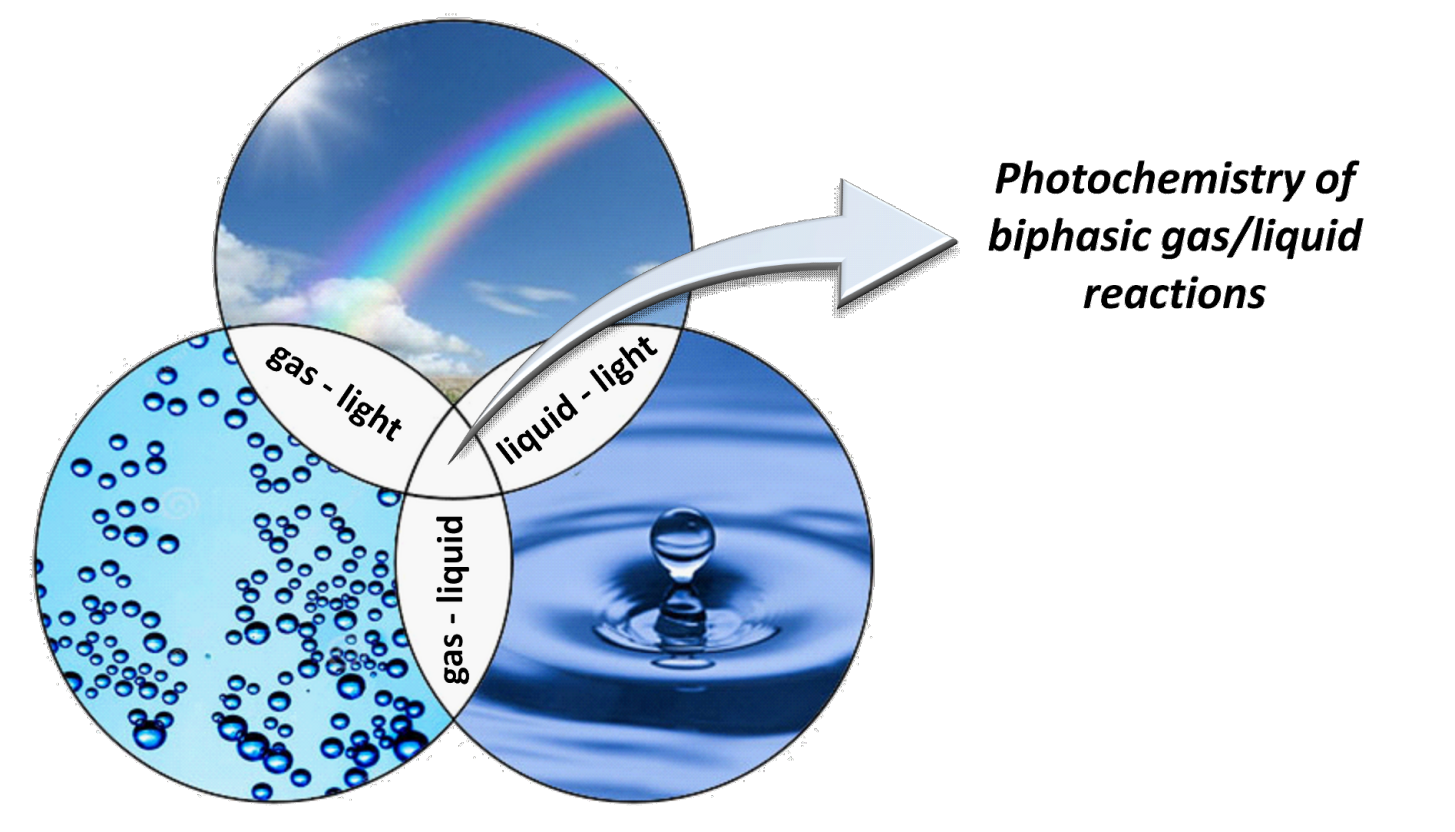
The challenge of mixing the three dispersed entities gas, liquid, and light for photochemical applications.

The vast majority of applications of microflow setups involved reagents in the same aggregation state (homogeneous, mostly liquid phase). In contrast, an especially complex problem beyond the scope of most microreactor setups are heterogeneous reactions [31–32] between three dispersed entities such as a liquid phase, a gas phase, and an electromagnetic radiation field, where an effective interaction of three quasi mobile phases of different physical states is required for high selectivities and conversion rates. Such scenarios are highly relevant for photochemical reactions with reactive gases (e.g., air, O_2_, O_3_, H_2_, Cl_2_, acetylene, NO_x_, CO, CO_2_, etc.) but obviously bear several challenges with regard to a reproducible and precise control of the addition and mixing of the three phases: the liquid phase, containing organic substrates, the photo-active component (catalyst or sensitizer), the solvent, and possibly additives, the gas phase, containing the gaseous reagents, and the light (Figure 1).

Only very few microreactor setups for such “quasi tri-phasic” processes have been reported [33–34]; most of them are film/falling film [22], microchannel [23,35] or simple tube reactors [35–37]. The reactor reported herein differs from most of the known systems in some key characteristics. Small-dimensioned thin film/falling film and microchannel reactors allow residence times in the seconds-to-few-minute range, which holds great potential for rapid conversions but is unsuitable for the significantly slower rates of many common organic reactions. Additionally, the gas-permeable tubes for tube-in-tube reactors and the photo-lithographically etched [38] microchannel plates are highly sensitive and expensive parts which limit their use by the average organic lab chemist. For comparison, the home-built reactor detailed in this report reliably operates at much longer residence times (up to 30 min) and uses cheap yet robust FEP (fluorinated ethylene propylene) tubing as transparent reactor material. While film/falling film and microchannel reactors display excellent mass-transfer and irradiation properties in several cases [39], our reactor shows higher versatility and tunability at a much lower price. In the following, we detail the technical specifications, step-by-step assembly, and lab-scale operation of an affordable, robust, and modular home-made flow reactor which shows great promise for general applications to photochemical reactions at gas/liquid interphases. Special attention has been paid to an especially simple reactor set-up which is based on cheap and available materials and parts, which can be assembled and operated with minimal technological expertise by students and researchers, yet is applicable to a wide range of reaction types. All parts of the modular reactor can be easily exchanged (capillaries, light source, pumps, mixer, valves, etc.) and the reagents widely varied up to 2 mL min^−1^ liquid reagents and 50 bar inlet pressure. The overall price of the whole system is below 10,000 €.

**Scheme 1:**
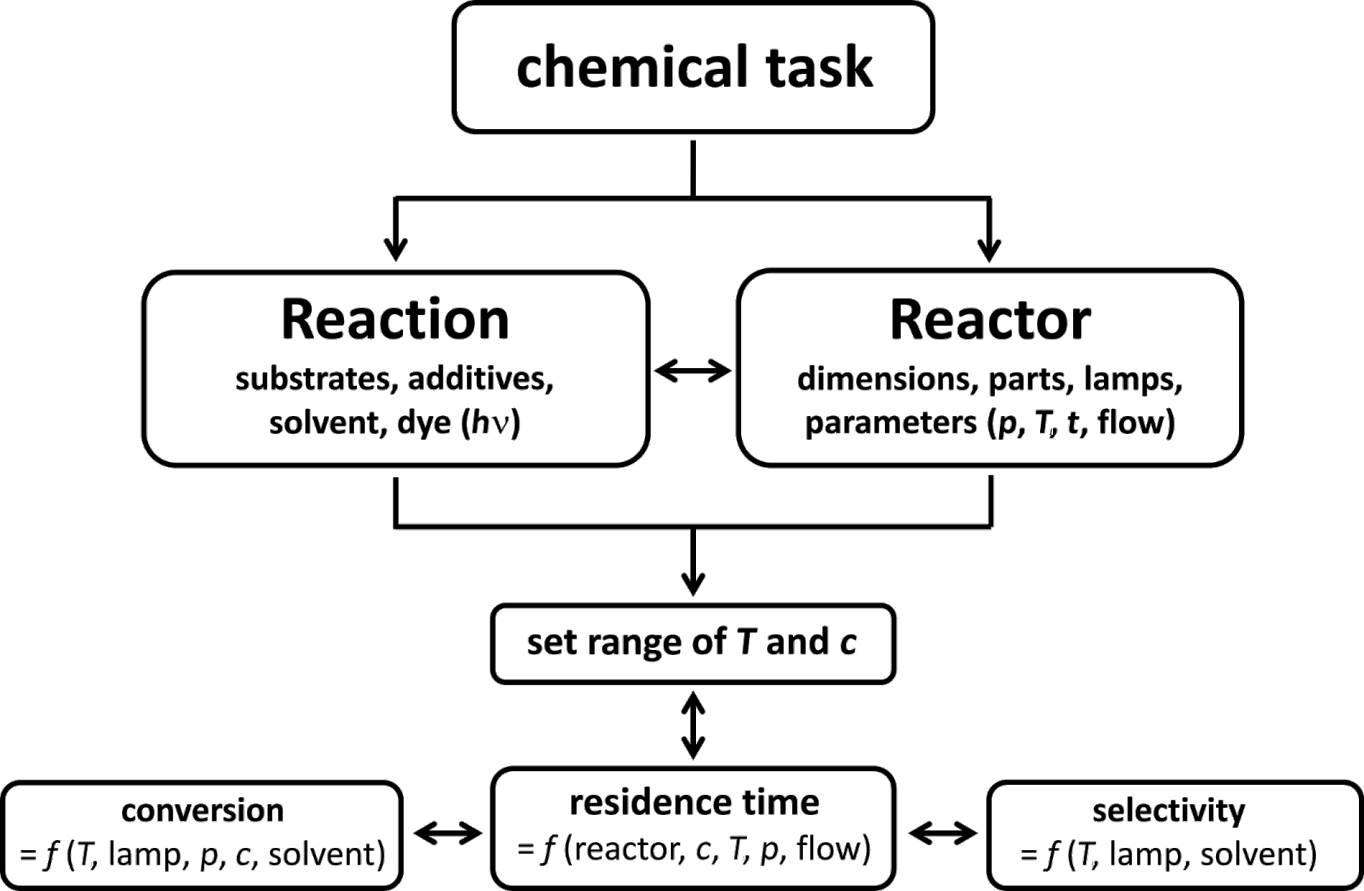
Mutual interdependencies of critical reaction and reactor parameters.

The wide variation of all three reaction components (liquid, gas, light) with regard to their nature (type of substrates, solvents, additives, gases, wavelength of light) and concentration (chemicals in solution, partial pressure of gas, light intensity) was a prime objective of this work. Furthermore, the technical parameters of the reaction and the reactor should be variable (temperature, pressure, flow rate, residence time, and size and type of tubing and reaction chamber). It is important to realize that most of these parameters cannot be varied individually but are mutually dependent on each other (e.g., choice of chromophore vs wavelength of light vs type of (transparent) reactor walls, concentration of reagents vs residence time for complete conversion vs length of reactor vs flow rate, etc.). The awareness of such multidimensional interdependencies is a prerequisite for the expedient optimization of a given chemical task by proper choice of the general reactor setup and wide variations of the critical reaction and reactor parameters (Scheme 1).

An efficient interaction of all three “reagents” will require careful adjustment of the light source, the wall material, the penetration length, the solution absorbance and other parameters. Until today, such protocols under flow conditions are most advanced with photosensitized oxidations which most recently have matured to great efficacy and versatility by the works of Seeberger et al. and Noël et al. Prominent examples include the photooxidations of citronellol [35,40–42], indanes [43], monoterpenes [36], furans [42], furfurals [44], thiols [37] and amines [45] as well as the syntheses of ascaridol [46] and artemisinin [47]. Related microreactor setups were applied to biphasic gas/liquid mixtures in the photochlorination of alkylbenzenes [48] and [2 + 2]-cycloadditions with ethylene [49].

## Results and Discussion

### Microreactor parts and setup

When studying the numerous literature reports of applications of flow reactors to organic synthesis it became obvious that there are no simple and quick technical solutions to such endeavours available to John Doe lab chemists who have no close collaboration with engineering specialists or do not wish to purchase sophisticated high-end devices for >10,000 Euros.

**Scheme 2:**
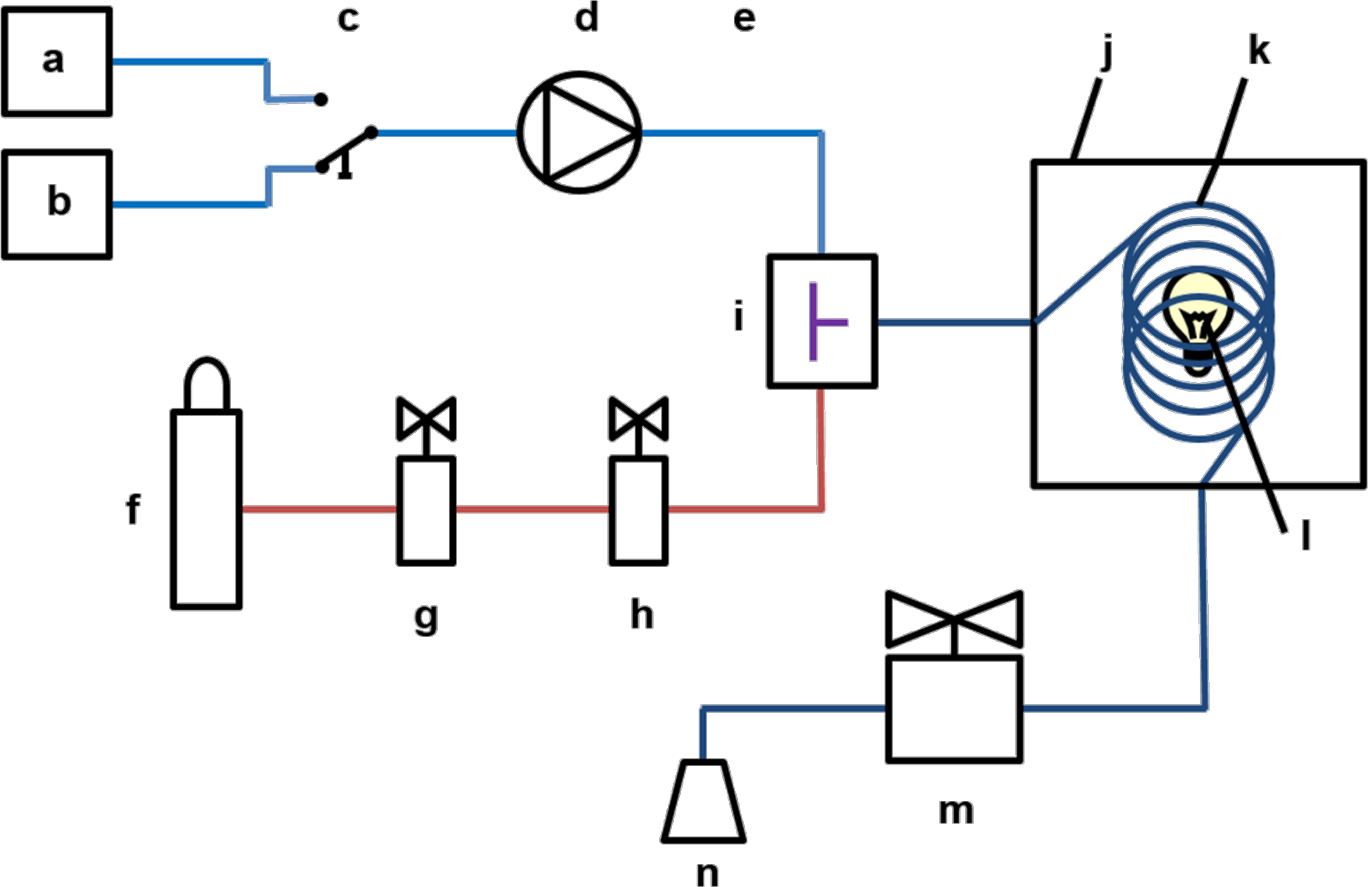
Blueprint of the home-built microflow photoreactor; schematic illustration of the reactor setup with a) solvent reservoir, b) substrate solution, c) T valve (BOLA, F 731-02), d) HPLC pump (Bischoff dosage pump 2250), e) capillary (for back-pressure build-up; Bischoff PEEK capillary 1021903PK), f) oxygen supply (Linde 4.6, 200 bar), g) pressure reducing valve *(*GO regulator, TeamTrade, PR1­1I11ACW-111), h) mass flow controller (Brooks SLA 5850), i) T mixer (IDEX, P-727 or P-632), j) thermostat bath, k) FEP capillary, l) LED light source (24 × Cree Xlamp MK-R, warm white, 700 mA), m) back-pressure regulator (IDEX, P-763, 100 psi), n) collecting vessel.

Stimulated by the ground-breaking works of the Seeberger [41–42,47,50–51], Booker-Milburn [52–53], and Noël [34,54–55] groups in recent years, we therefore decided to develop our own home-made flow reactor for photooxidations of organic molecules but also envisaged its general applicability to other challenging processes with the ternary “reagent” combinations liquid/gas/light. With the objective of constructing a robust and versatile flow reactor, we set out to explore and test commercially available parts. Major emphasis was placed on maximum flexibility with regard to reaction parameters (reagents, concentrations, temperature, reaction/residence time), and technical parameters (light sources, flow rates, pressure, reactor type, size and length). The continuous operation with reproducible results would require the use of components that ensure strictly constant gas and liquid flow rates, mixing properties, irradiation over the period of operation. In the following, we wish to provide a hands-on manual for the design and set-up of a flow reactor for photochemical reactions at gas/liquid interfaces in any standard student or research laboratory. The requirements, specifications, and pitfalls of the most critical technical components will be discussed from the perspective of a non-expert user (Scheme 2).

#### Solution pump

Standard syringe pumps generate significant pulsation so that HPLC compact pumps are much better suited for the continuous generation of a steady slug flow. However, HPLC pumps are in direct contact with the reaction medium and thus can experience degradation with reactive reagents/solvents. Special attention should be directed at the homogeneity of the liquid phase in case of, e.g., limited solubility or biphasic systems which might require the addition of a co-solvent, prior filtration of the solution phase, or the installation of an upstream filter. It is important to consider that most HPLC pumps only provide accurate and steady solution pumping when operating against a significantly high back-pressure from the column chromatography unit. Therefore, a very thin capillary (l = 6 m, internal d = 0.13 mm) was placed between the pump and the reactor. This capillary generates significant back-pressures at low flow rates (16 bar/32 bar in acetonitrile and 27 bar/54 bar in methanol at 0.5/1.0 mL min^−1^, respectively) and ensures a pulsation-free operation of the pump.

#### Pressure reducing valve & manometer

As with the pumps, the pressure reducing valve has to ensure a constant pressure regime during the whole span of the reaction time. Upon careful consideration and multiple tests, we have opted for a GO regulator prepared for oxygen usage, which covers the targeted flow rates (up to 25 mL min^−1^) and pressure range (52 bar maximum outlet pressure). An erratic operation of the pressure reducing valve causes pressure spikes which prohibits a steady volume stream through the mass flow controller.

#### Mass flow controller

The mass flow controller fulfills an identical role as its liquid phase complement, the pulsation-free pump. The precise control of the mass flow of the gaseous reagents (here oxygen) ensures a constant gas stream which directly correlates with the build-up of a constant slug flow and a reproducible and constant productivity (conversion per time interval) and thereby allows the continuous operation of the flow reactor. Unlike the so-called gas flow meters, mass flow controllers are active devices that constantly measure and adjust the current volume stream to the target value. It is especially important that the mass flow controller is sufficiently pressure-resistant for the specific gases and pressure range used and precisely calibrated. As aforementioned for the HPLC pumps, thin-capillary reactors can build up large back-pressures. The initial upstream entry pressure should be set rather high in order to accommodate the significant pressure gradient over the capillary length.

#### T mixer

There are numerous types of mixers for various purposes. However, simple T-valves are suitable for most applications. A low dead volume and a high pressure resistance are key characteristics of T mixers. However, careful optimization of the internal size, shape, and dead volume of the T-mixer directly affects the local concentrations of the reagents and thus can have dramatic effects on reaction rates and selectivities. Furthermore, specific properties of the reagents and solvents (viscosity, surface tension, gas solubility) will be critical to the dispersion and slug flow formation.

#### Light source

The benefit of microreaction technologies applied to photochemical processes is especially evident from a consideration of the extent of light attenuation when passing through condensed matter. The molar attenuation coefficients of common organic photosensitizers are in the range of 20,000–500,000 M^−1^ cm^−1^ (Table 1) which significantly limits the penetration depth of visible light into standard batch reactions at 10^−4^–10^−2^ M concentrations of the dye so that large volumes of the reaction remain in the dark [9,52].

**Table 1:** Molar attenuation coefficients of common photosensitizers^a^ (bpy = 2,2‘-bipyridine).

Sensitizer	Molar attenuation coefficient ε [M^−1^ cm^−1^]

rose bengal disodium salt	112,400 [56] (in ethanol)
methylene blue	85,100 [57] (in acetonitrile)
tetraphenylporphyrin	478,000 [58] (in chloroform)
sodium fluorescein	76,000 [59] (in water, pH 7.4)
[Ru(bpy)_3_]Cl_2_	14,600 [60] (in water)

^a^Maximum attenuation coefficients in the visible absorbance spectrum of the mentioned photosensitizers in the respective solvent.

**Figure 2:**
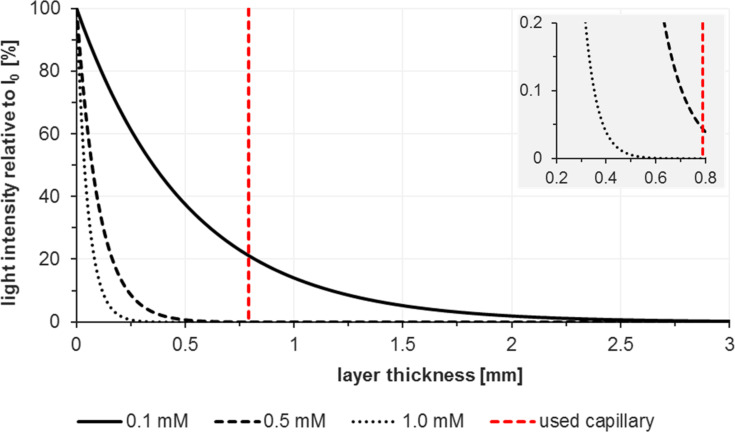
Total absorbance of methylene blue solutions in acetonitrile according to the Beer-Lambert law: *E*_λ_ = ε_λ_·c·d. [63]. The diameter of the applied capillary, 1/32 inch or 0.79 mm, is shown in red.

It is important to note that the magnitude of attenuation coefficients of the employed chromophores is a key difference between the recently emerging field of photocatalysis (with dyes of ε > 10,000 M^−1^ cm^−1^) and traditional photochemistry (involving mostly UV irradiation of colourless organic molecules of ε < 1,000 M^−1^ cm^−1^) [61]. The much lower molar attenuation coefficients of the majority of organic molecules which are directly irradiated by UV light in photochemical processes (e.g., *N*-alkyl maleimides, ε = ~700 M^−1^ cm^−1^) [62] lead to more efficient light penetration in batch reactions and therefore only show a limited benefit of using flow reactors for such purposes. On the other hand, a 1 mM solution of methylene blue exhibits total absorption (>99.9%) at 0.35 mm penetration depth in a solution of acetonitrile (Figure 2) [63]. The high surface-to-volume ratio of microreactors thus increases the relative pathway of light through the solution, speeds up the rate of reactions, and minimizes competing side reactions [11,52,64–65]. Total light absorption of dye solutions with high attenuation coefficients can be prevented by low-diameter reactor dimensions and flow conditions that favour the formation of thin films along the reactor walls.

**Figure 3:**
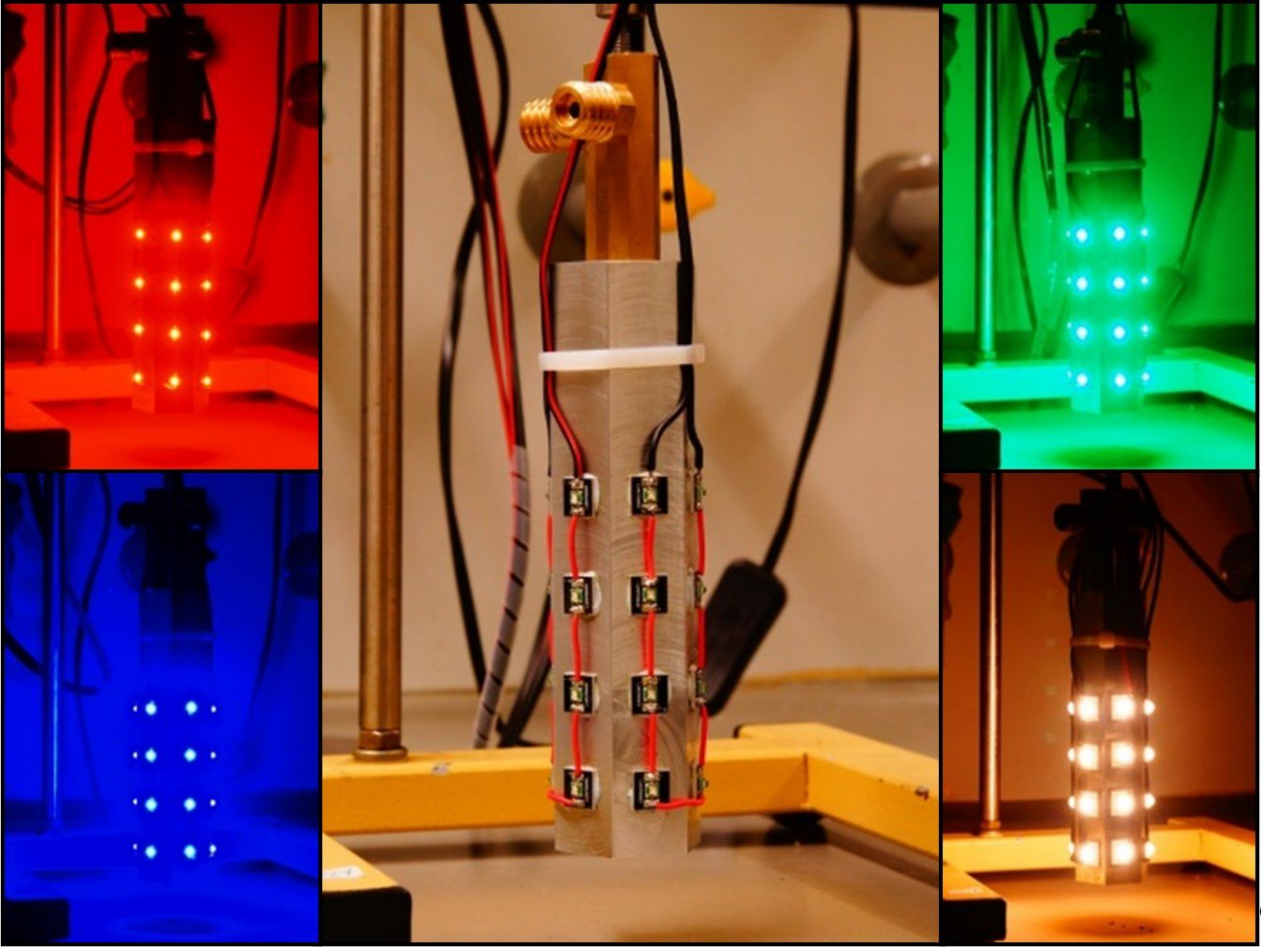
Red (λ_max_ = 633 nm), blue (λ_max_ = 448 nm), green (λ_max_ = 520 nm) and white (λ_max_ = 620 nm) LEDs mounted on a water cooled aluminium cooling element.

**Figure 4:**
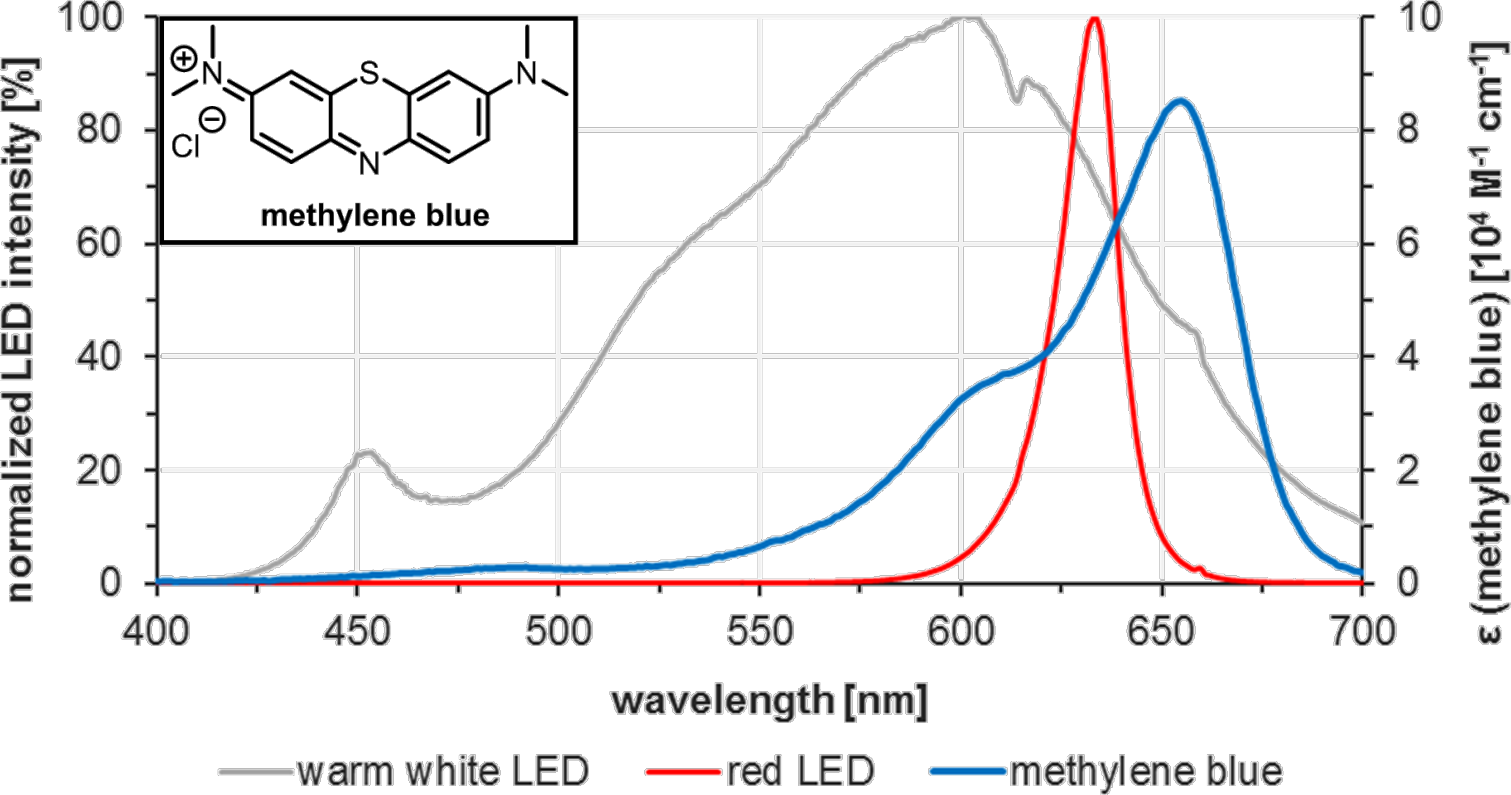
Overlap of absorption spectrum of methylene blue in acetonitrile and emission spectra of reasonably overlapping light sources.

**Figure 5:**
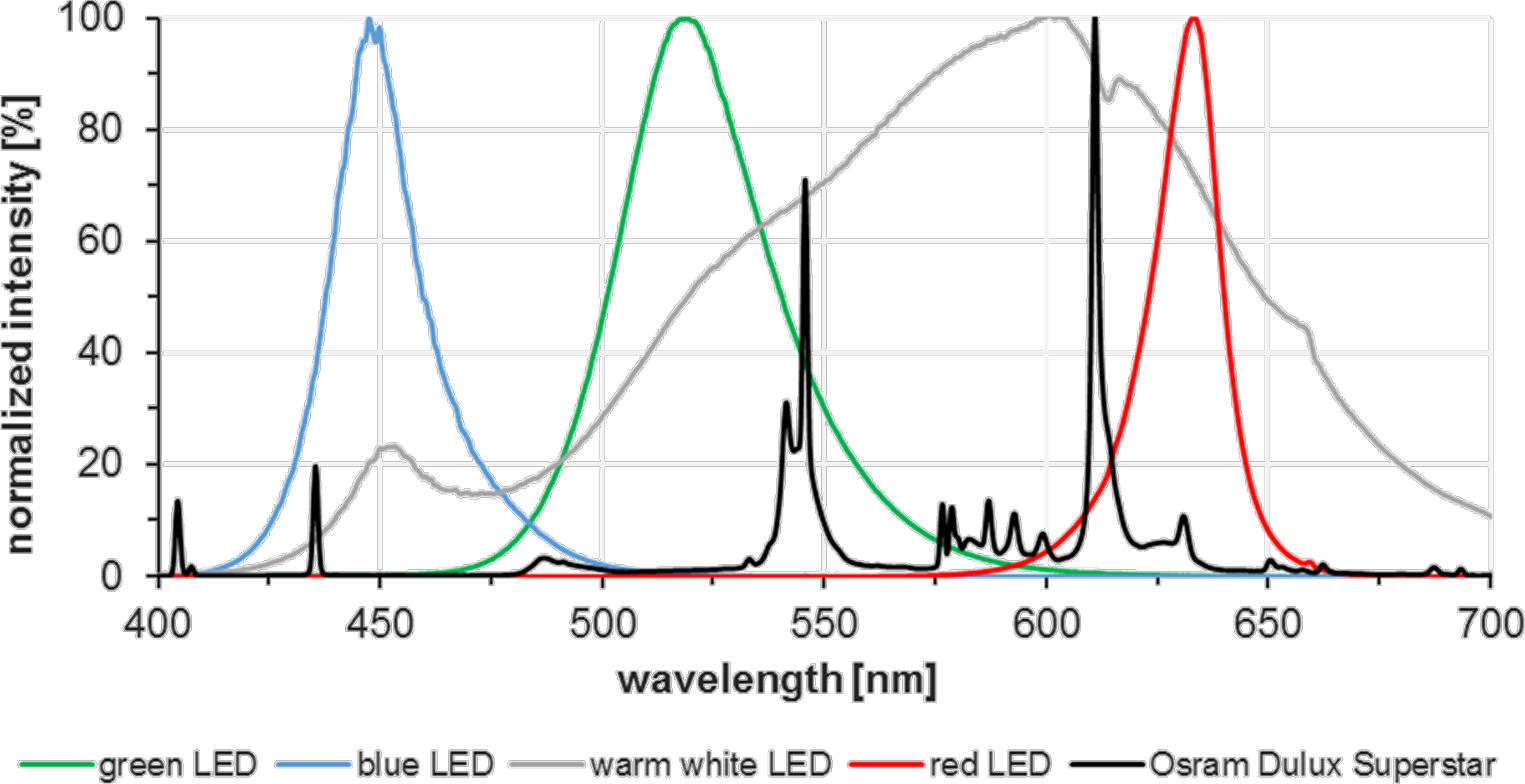
Emission spectra of different LEDs; red (λ_max_ = 633 nm), blue (λ_max_ = 448 nm), green (λ_max_ = 520 nm), warm white (λ_max_ = 600 nm), and Osram Dulux Superstar.

Energy-saving light bulbs and light-emitting diodes (LEDs) of different wavelengths (red, green, blue, white) were used for irradiation of the reactions. The light source was placed in the center of the reactor to achieve optimal irradiation [66]. Key characteristics are the emission spectrum and the light intensity. A perfect match of the light emission maximum and the dye’s absorption maximum is desirable. It should also be considered that potential excitation of other reagents could trigger competitive reaction mechanisms and pathways. The LEDs were mounted on an aluminium rod which is water-cooled from the interior to prevent (over-)heating of the LEDs and the reaction (Figure 3). White light was obtained from a commercial energy-saving light bulb (Osram Dulux Superstar). LEDs exhibit high light power (~110 lm W^−1^) at low energy consumption in comparison with other powerful light sources such as mercury lamps (~50 lm W^−1^). The availability of various LED types with different wavelengths and narrow emission spectra obviates the need for filters and allows high quantum yields by the correct matching of lamp emission and chromophore absorption bands (Figure 4 and Figure 5). The absence of UV emission bands regarding the used red and warm white LEDs enhances the selectivity of many organic reactions by suppression of unwanted UV-mediated degradation processes.

#### Capillary tubing

**Scheme 3:**
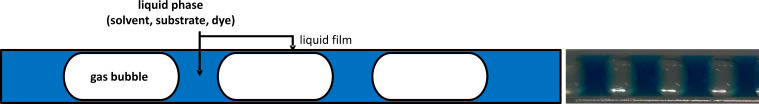
Slug flow conditions of two-phase gas-liquid mixtures. Photograph of a slug flow of a solution of methylene blue (1.0 mM), a cyclohexene (0.1 M), oxygen at 30 bar, and 1.5 mL min^−1^ flow rate in an FEP tubing reactor (0.79 mm inner diameter).

**Figure 6:**
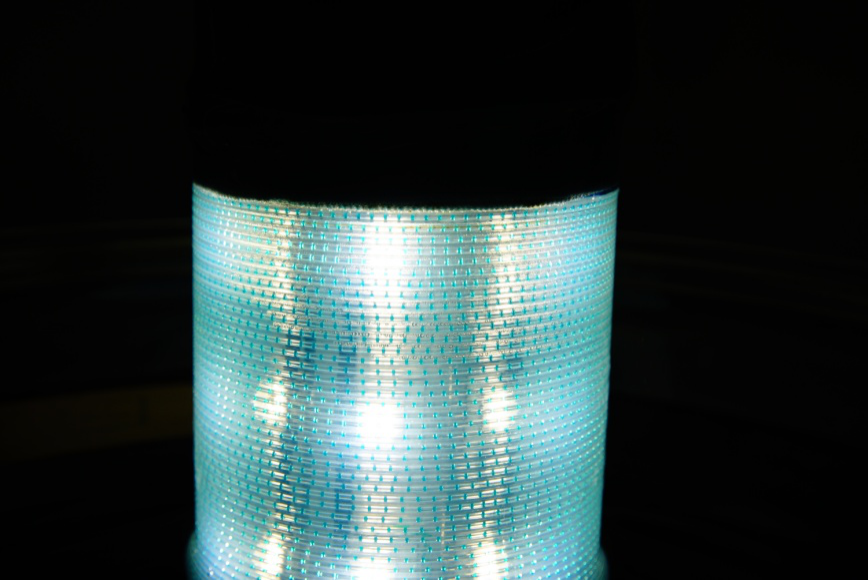
Photograph of the operating flow reactor, irradiated with white LEDs, filled with a solution of methylene blue (1.0 mM), a cyclohexene (0.1 M), and oxygen at 30 bar with a flow rate of 1.0 mL min^−1^ in an FEP tubing reactor (0.79 mm inner diameter).

With gas-liquid two-phase flows, several flow regimes can form which mostly depend on the configuration of the inlets, the gravity, the size of the tube, the fluid properties, and the flow rate. A steady and highly dispersed two-phase flow is present in the so-called slug flow (Scheme 3, Figure 6). The resultant thin film around the gas bubbles and along the reactor walls allows large portions of the substrate solution to be efficiently irradiated while being at the same time in contact with the gas phase. This leads to strongly enhanced mass transfer coefficients compared to traditional stirred tanks [39]. Depending on the flow rates of gas and liquid phases, their ratio, the solvent viscosity and the reactor dimensions, different flow pattern can be obtained. The so-called slug flow leads to very efficient irradiation but also other spatial distributions (plug flow, bubbly flow, annular flow or isolated gas and liquid segments) can occur under certain conditions (Scheme 3, Figure 6) [67].

The formation of a steady slug flow can be best achieved in a long tubular reactor which leads to very efficient irradiation (Figure 6). Mixing of liquid phase and gas phase occurs upstream in a T mixer, while the tubular reactor should provide a high surface area for maximum exposure to the light source. For reaction times in the range of 10 s to 20 min, commercial fluorinated ethylene-propylene (FEP)-capillaries with an internal diameter of less than 1 mm (here 0.79 mm) were used and coiled around a glass cylinder containing the light source. It is again important to note that the tubing diameter can significantly influence the formation of the slug flow, the magnitude of light absorption, and the back-pressure build-up, which is again dependent on the tubing length and the specification of the back-pressure regulator. The choice of an FEP coil around the lamp allows maximal versatility with regard to the type of light source, the wall material, the size and length of the tubing and thus allows multidimensional tuning of some of the most important reactor parameters. The length of the tubing coil directly determines the residence time in the reactor. The FEP tubing does not exhibit a significant absorption of visible light [68]; furthermore, the reaction temperature can be easily set and controlled by immersing the reactor coil into a thermostat bath. This set-up is advantageous as the cooling liquid does not absorb any of the incident light before it penetrates the reactor and the outer cooling counters the heat generated by the central lamp. FEP-capillaries for HPLC applications are available in various internal and external diameters. The optimal tubing gauge of photoreactors allows irradiation of the whole reactor width, which is determined by the Beer–Lambert law. The light intensity within the reactor is dependent on the concentration and absorption coefficient of the absorbing materials (reactor walls, dye, etc.).

**Scheme 4:**
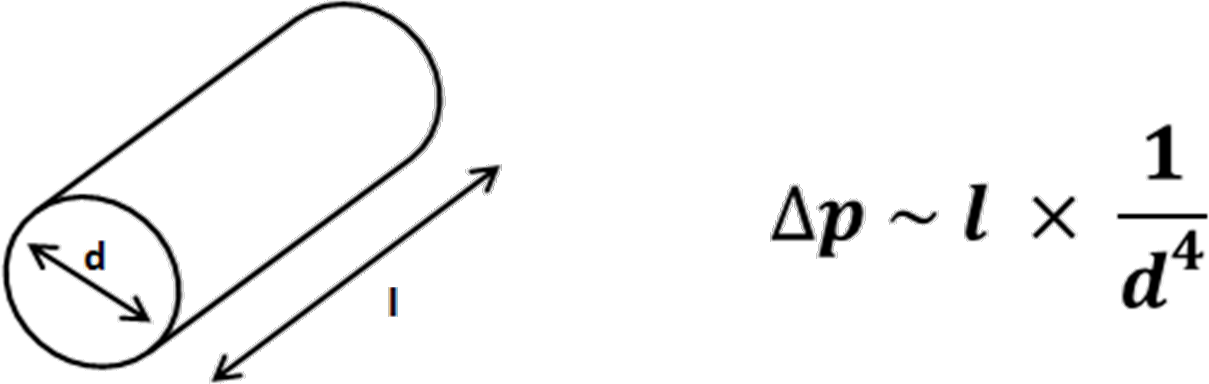
Schematic illustration of a reactor tube (length l, inner diameter d) and pressure gradient Δp according to the Hagen–Poiseuille law.

Generally, the used capillaries should be of high quality to resist up to the required pressures (approx. 35 bar for a 30 m tube, with an inner diameter of 0.79 mm) without significant expansion of the inner size, which would cause variable internal pressures and perturb the slug flow. The back-pressure generated by the capillary tubing is dependent on the length and gauge of the capillary and the flow rate of the solution. If the dynamic viscosity of the solution is known, the pressure gradient can be calculated from the Hagen–Poiseuille equation (Scheme 4).

#### Temperature control

The microreactor system is equipped with a dual water cooling. The LED rod has an interior active water cooling. The reaction tubing is immersed into a water/ethanol bath which is controlled by an external thermostat. This setup avoids that the light has to pass through the cooling media as in double-walled reaction vessels (absorption).

#### Back-pressure regulator

This device fulfills many roles: It controls the pressure at the downstream end of the reactor which should be greater than the ambient pressure in order to avoid both, the increasing expansion of the gas bubbles and the exceeding increase of the flow rate over the length of the reactor. A sufficiently high back-pressure secures a constant flow throughout the length of the tubing. This set back-pressure has to be higher than the pressure drop within the capillary which is especially high in long and thin capillaries. The back-pressure regulator also controls the solubility of the gas in the liquid phase which is pressure-dependent according to Henry’s law [69].

#### Reaction parameters of a model photooxygenation

**Scheme 5:**
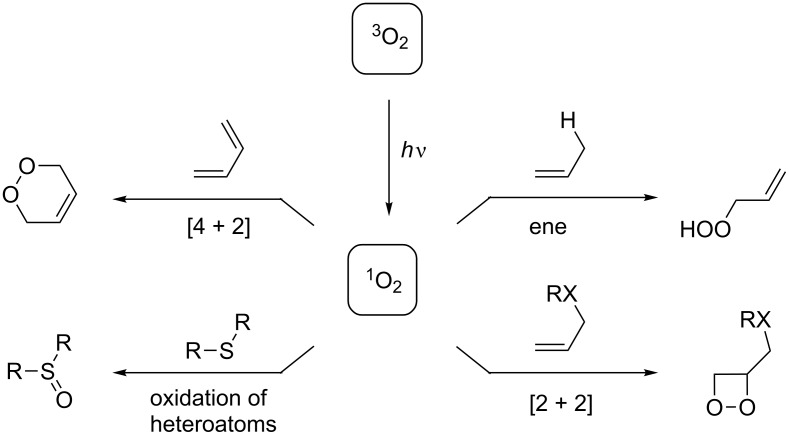
Reaction types of organic molecules with singlet oxygen.

The often poor selectivities of reactions with molecular oxygen (being a triplet biradical in its ground state) [70] have prompted applications of microflow reactors to selective oxidations of various organic molecules (Scheme 5). From a conceptual point of view, the combination of two of the most abundant “reagents” on the surface of our planet, oxygen and visible light, with a safe, scalable, and efficient reactor technology for chemical reactions constitutes an approach to oxidation chemistry of utmost sustainability. Under irradiation in the presence of a sensitizer, singlet oxygen can easily be generated from the triplet ground state. Several applications of such photooxidations to chemical synthesis have been reported [71–73], in recent years most effectively under microflow conditions [41–42,50–51,74–75].

**Figure 7:**
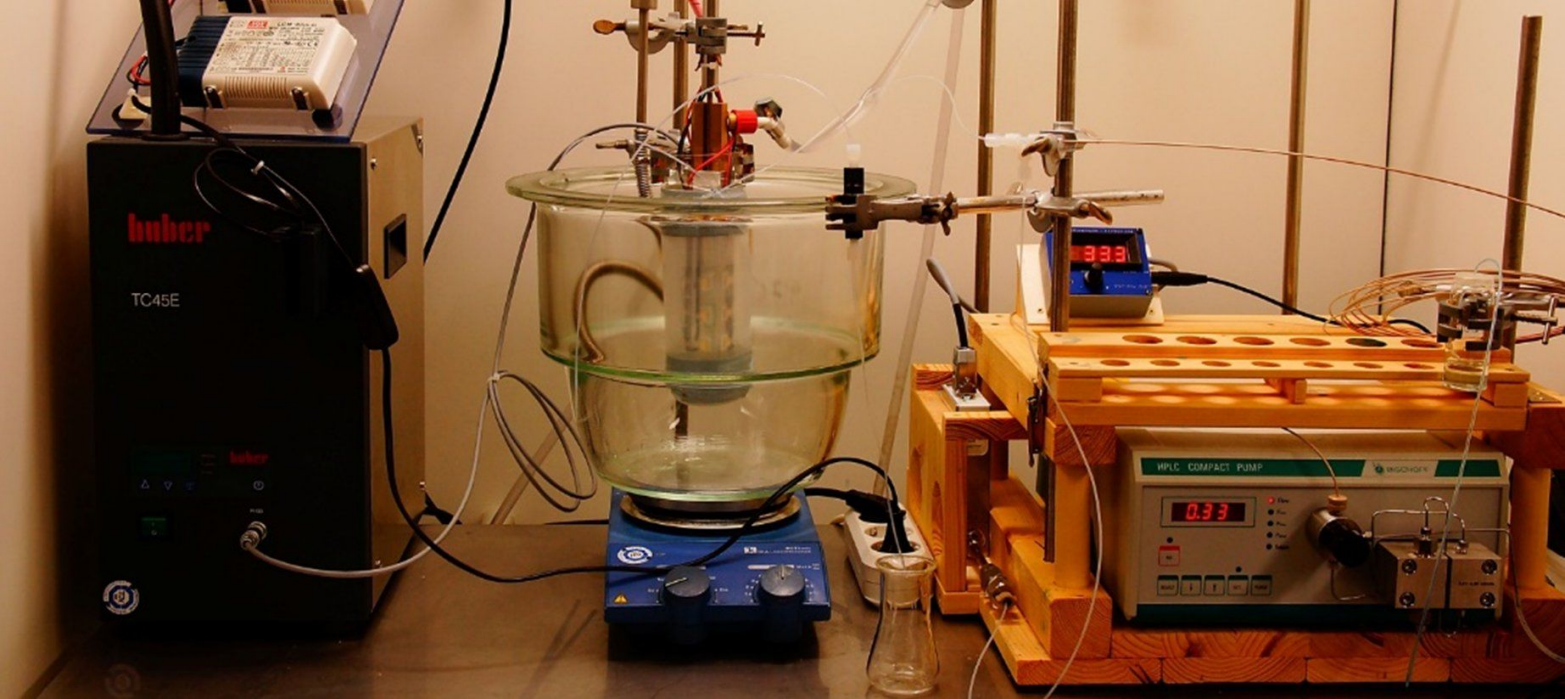
Home-made flow reactor and peripheral devices for photochemical reactions at light/liquid/gas interfaces.

**Scheme 6:**
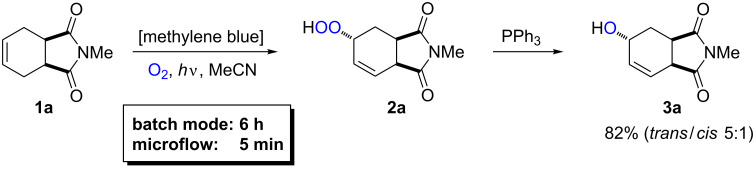
Photooxygenation of *N*-methyl-1,2,3,6-tetrahydrophthalimide and reductive work-up to alcohol **3a**.

We applied the home-made photo-flow reactor (Figure 7) to the visible light mediated oxygenation of a cyclohexene derivative (i.e., Schenck ene reaction with singlet oxygen) [72–73] and evaluated the critical reaction and reactor parameters. Following an optimized Schenck ene reaction procedure, *N*-methyl-1,2,3,6-tetrahydrophthalimide (**1a**) was reacted with molecular oxygen in the presence of methylene blue as sensitizer (Scheme 6). The resultant hydroperoxide motif (**2a**) constitutes a valuable carbocyclic building block. For reasons of convenience, reductive work-up with triphenylphosphine (PPh_3_) was performed to obtain the stable allyl alcohol derivative **3a** which offers ample opportunities for chemical manipulations at the alcohol, alkene, and carboxamide functions. The choice of solvent is crucial as it determines the solubility of the organic substrate and the dye, and the solubility as well as the lifetime of oxygen in its singlet state (^1^O_2_) [76]. All reactions were performed under irradiation with an energy-saving light bulb (Osram Dulux Superstar) against a back-pressure of 2.4 bar while one parameter was varied in each experiment.

It is especially important to note that reactions of non-activated substrates which require long residence times and low flow rates display strong interdependencies of reaction parameters which are often negligible at higher flow rates [41–42,50–51]. While higher substrate concentrations showed only slightly higher conversions, a significant increase of residence time at constant flow rates was observed (Table 2). This can be attributed to the increased O_2_ consumption at higher substrate concentrations and the resultant reduction of gas volume and flow rate over the capillary length. At identical residence times, the conversion of **1a** could only be slightly improved with lower concentrations (after 6.75 min: 80% with 0.20 M, 87% with 0.01 M). This results in an overall dramatic increase of reaction productivity at higher substrate concentrations. In order to minimize the effect of substrate concentration on the flow rates, the internal reactor pressure must be increased which leads to a higher O_2_ density, lower relative O_2_ consumptions, and shorter residence times. Ongoing experiments with an inlet pressure of 35 bar do however only show insignificantly longer residence times for higher substrate concentrations.

**Table 2:** Conversion vs substrate concentration.^a^

Substrate [mol L^−1^]	Residence time [min]	Conversion [%]	Productivity [µmol min^−1^]

0.01	5.00	75	1.9
0.05	5.50	70	8.8
0.10	6.25	75	18.8
0.20	6.75	80 (87^b^)	40.0
0.30	8.00	90	67.5
0.40	7.70	84	84.0
0.50	10.5	89 (95^b^)	111.3

^a^Reactions at constant flow rates in acetonitrile at 10 °C; 1 mM methylene blue, 2.4 bar back-pressure, Osram Dulux Superstar. Conversions were determined by quantitative GC-FID vs internal standard dodecanenitrile. ^b^Conversion at identical residence time and 0.01 M substrate solution.

**Figure 8:**
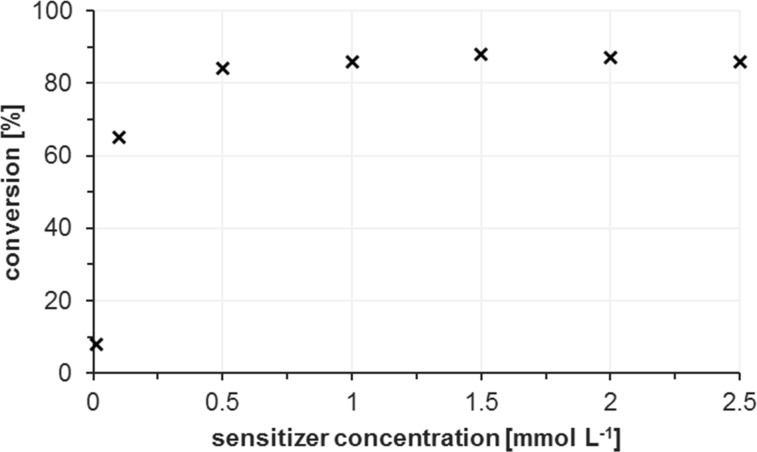
Conversion vs methylene blue sensitizer concentration. Reactions at constant flow rates in acetonitrile at 10 °C; 0.01 M **1a**, 2.4 bar, light source: Osram Dulux Superstar.

A similar effect on the residence time was observed by variation of the reaction temperature. Gas-phase compression at higher temperature leads to shorter reaction times while maximal conversion and productivity were achieved at room temperature. A more direct relationship between conversion and residence time resulted from variations of the concentration of the sensitizer methylene blue (Figure 8). At >0.5 mmol L^−1^, a plateau was reached (at very high dye concentrations, >2.0 mmol L^−1^, ^1^O_2_ quenching leads to lower conversions) [43,77].

Variations of the used light source regarding power and wavelength show the importance of the conformance of the absorption spectrum of the sensitizer and the emission spectrum of the light source. Table 3 illustrates the influence of the irradiation wavelength and intensity on the conversion at different residence times.

**Table 3:** Residence time and conversion with different light sources.^a^

Light source	Residence time [min]	Conversion [%]

Energy-saving lamp (white)	6.25	75
LED (white)	1.50	87
LED (white)	3.50	>99
LED (red)	3.50	80
LED (red)	6.25	92
LED (red)	8.00	>99

^a^The reactions were performed in acetonitrile at 10 °C with concentrations of 0.1 M **1a**, 1 mM methylene blue, and a back-pressure of 2.4 bar (inlet pressure 10 bar)*.* The conversions of the photooxidation were determined by quantitative GC/FID vs internal standard dodecanenitrile.

**Figure 9:**
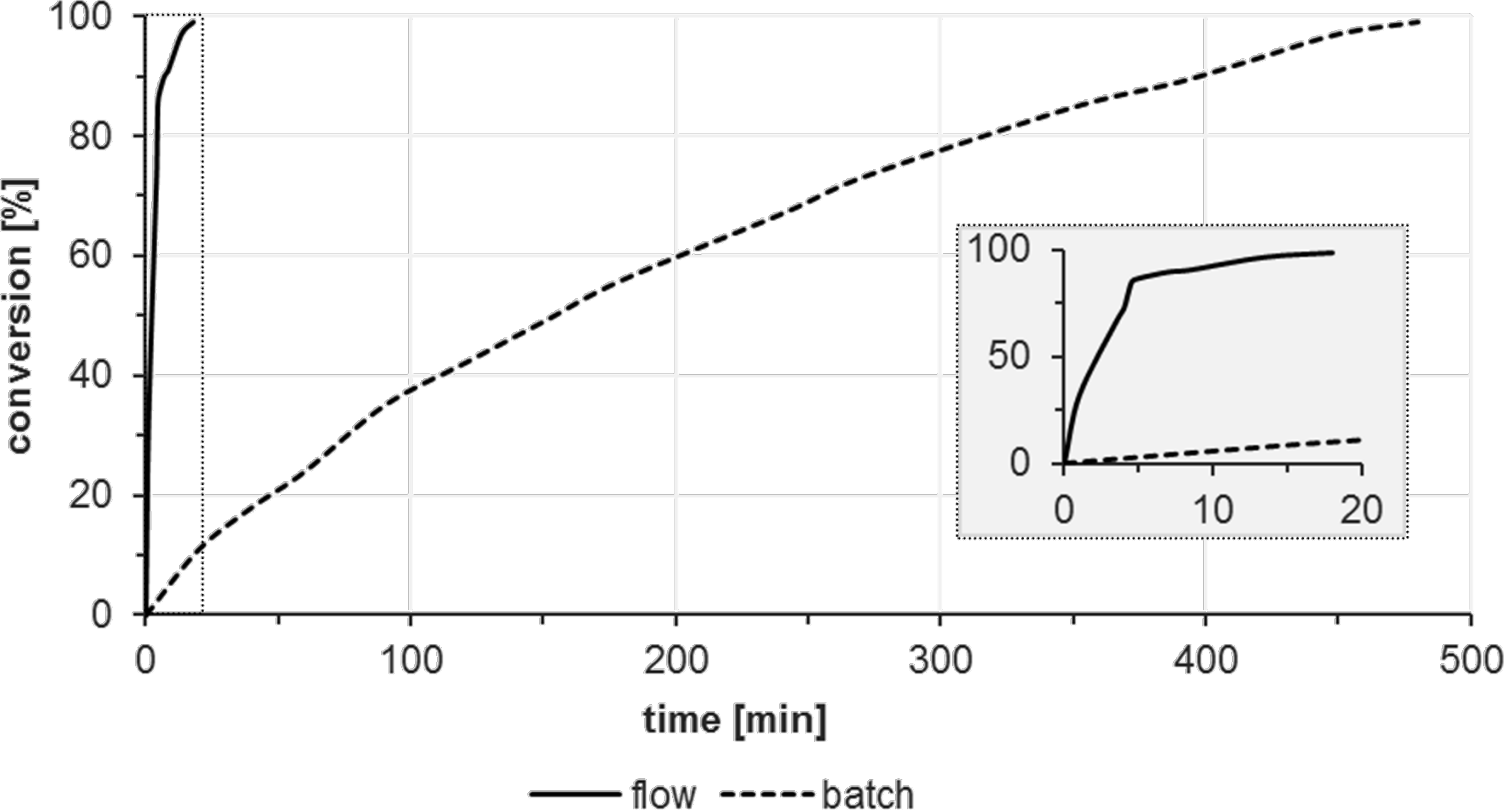
Reaction progress at different residence times in flow and batch reactions. Flow: reactions at different flow rates in MeCN at 10 °C; 0.01 M **1a**, 1 mM methylene blue, 2.4 bar, irradiation by Osram Dulux Superstar. Batch: 0.05 M **1a**, 0.1 mM methylene blue in MeCN at rt, irradiation by warm white LEDs.

A direct relation between residence time and conversion is illustrated in Figure 9. Quantitative conversions were reached after 13.5 min; shorter residence led to lower conversion. The flow reactor assured full conversion after several minutes for which the batch reactions required 8 h. Furthermore, the residence time can generally be modulated by the length of the reactor tubing and the flow rates of solution and gas phases.

**Scheme 7:**
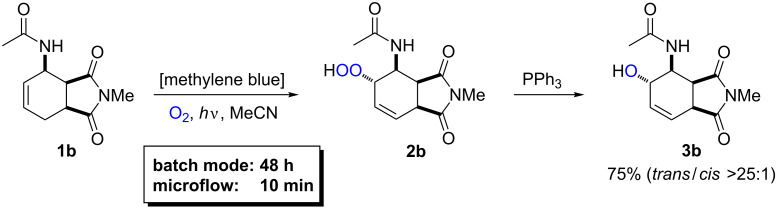
Oxidation of *N*-methyl-1,2,3,6-tetrahydro-3-acetamidophthalimide and reductive work-up to alcohol **3b**.

A comparison of the utilized light power of identical LEDs which is required for complete conversion of 1 mmol substrate in batch and flow documents that the six LEDs of the batch reactions have consumed 0.56 kWh electricity within 8 h. This equals the power of the 24 LEDs of the flow reactor after 2 h operation which converts 9 mmol of substrate (0.062 kWh mmol^−1^). An even more pronounced advantage of the flow reactor becomes evident when using less reactive starting materials (Scheme 7). The aminocyclohexene derivative **1b** [78–81] was quantitatively converted to hydroperoxide **2b** which displayed perfect *trans*-diastereocontrol. After 48 h in batch mode, 3.37 kWh were consumed per mmol starting material, which in flow mode would suffice for continuous conversion of 36 mmol substrate over 12 h at 0.5 mL min^−1^ flow rate (0.09 kWh mmol^−1^).

## Conclusion

The basic technological and practical aspects of a home-made microreactor setup for applications to photochemical processes with gas/liquid mixtures have been described in full detail. Special care should be taken with the design of the key components (pumps, tubing, pressure valves) and the key parameters (tube diameter, tube length, back pressure, flow rate) and with their mutual interdependencies. We have presented a thorough analysis of critical parts and conditions of a general microflow reactor setup for photochemical reactions and an exemplary application to oxidations with gaseous oxygen as the stoichiometric oxidant. The range of application of such a modular home-built reactor is 0.04–0.5 mmol min^−1^ substrate throughput, 0.4–2.5 mL min^−1^ liquid phase flow rates, −40 to 60 °C, and 5–40 bar oxygen pressure. The device can be easily assembled within one day from commercial parts at an overall price of less than 1000 € for the microreactor, less than 5000 € for the peripheral pump and mass flow controller, and less than 500 € for each light source coil. The application of this microreactor system to the visible light-driven photooxygenation of cyclohexene derivatives documented the superiority over a standard batch process in terms of productivity. Depending on the solubility of the substrates up to 30 mmol h^−1^ could be oxidized.

We believe that this report provides a stepping stone for researchers, teachers, and students around the world who wish to enter the field of microreactor technology for organic reactions at a reasonable price and effort. This detailed report on the theoretical, technical, and chemical aspects of a non-expert application of a home-built microreactor to a standard chemical reaction is specifically intended to stimulate multiple reproduction.

## Supporting Information

File 1 Experimental.
