# Supplementary material for: A flow reactor setup for photochemistry of biphasic gas/liquid reactions
Source: Beilstein J Org Chem. 2016 Aug 11;12:1798–811. doi: 10.3762/bjoc.12.170 (PMC5082722; doi:10.3762/bjoc.12.170)
Supplement: File 1 — Experimental. [file Beilstein_J_Org_Chem-12-1798-s001.pdf]

Supporting Information

for

A flow reactor setup for photochemistry of biphasic gas/liquid reactions

Josef Schachtner, Patrick Bayer and Axel Jacobi von Wangelin*

Address: Institute of Organic Chemistry, University of Regensburg, Universitaetsstr. 31, 93040 Regensburg, Germany

Email: Axel Jacobi von Wangelin - axel.jacobi@ur.de

* Corresponding author

Experimental

General

Commercial chemicals (≥98% purity) were used as obtained without further purification. TLC was performed using commercial silica gel coated aluminum plates (DC Kieselgel 60 F_{254}, Merck); visualization was done by the use of UV light. Staining was realized with a solution of phosphomolybdic acid or potassium permanganate in ethanol. Visualization was done by UV light. Product yields were determined from isolated materials after normal or flash column chromatography on silica gel (Acros Organics, mesh 35–70) or for optimization and screening purposes by quantitative GC-FID measurements, on an Agilent 7820A GC-System with N_2 as carrier gas. Low-resolution mass detection was carried out on an Agilent 6890N GC-System with 5975 MS mass detector and H_2 as carrier gas. Infrared spectra were recorded on an Agilent Cary 630 FTIR spectrometer equipped with an ATR unit. Intensive absorption bands are indicated with s (strong), medium bands with m (medium), and weak bands with w (weak). Purity and structure confirmation was performed by ^{1}H NMR, ^{13}C NMR, and MS. Abbreviations used in MS spectra: M – molar mass of target compound, r.I. – relative intensity. High resolution mass spectra were recorded by the Central Analytics at the department of chemistry, University of Regensburg, on various machines. NMR spectral data was collected on a Bruker Avance 300 (300 MHz for ^{1}H; 75 MHz for ^{13}C) spectrometer and a Bruker Avance 400 (400 MHz for ^{1}H; 101 MHz for ^{13}C) spectrometer at 25 °C. Solvent residual peaks were used as internal standard for all NMR measurements. The quantification of ^{1}H cores was obtained by integrations of resonance signals. Abbreviations used in NMR spectra: s – singlet, d – doublet, t – triplet, q – quartet, m – multiplet, bs – broad singlet, dd – doublet of doublet, ddd – doublet of doublet of doublet, R – organic rest, not hydrogen.

Synthesis of *N*-methyl-1,2,3,6-tetrahydophthalimide[1] (**1a**)

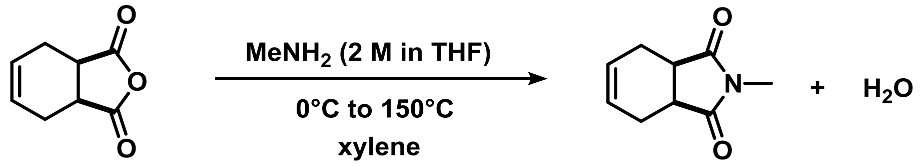

$C_9H_{11}NO_2$

152.15 **165.19**

Cis-1,2,3,6-tetrahydrophthalic anhydride (7.5 g, 49 mmol, 1.0 equiv) was dissolved in xylene (27 mL) and cooled to 0 °C. Methylamine (2 M in THF, 27 mL, 54 mmol, 1.1 equiv) was added at 0 °C. The reaction mixture was allowed to warm to rt and stirred at rt for 2 h. Subsequently, the suspension was stirred for 4 h at 150 °C using a water separator. The solvent was removed under reduced pressure and the crude product was purified by column chromatography using PE/EA (2:1) as eluent. **1a** (5.7 g, 70%) was isolated as colorless crystals.

TLC: R_f = 0.42 (PE/EA = 2/1)

^{1}H-NMR: (400 MHz, $CDCl_3$) δ [ppm] = 5.92-5.85 (m, 2H, CH-1,2), 3.12-3.05 (m, 2H, CH-4,6), 2.96 (s, 3H, CH-5), 2.64-2.58 (m, 2H, CH-3), 2.27-2.19 (m, 2H, CH-7).

GC-MS: (m/z): 165 [M^+]

Synthesis of *N*-(2-methyl-1,3-dioxo-2,3,3a,4,7,7a-hexahydro-1*H*-isoindol-4-yl) acetamide[2] (**1b**)

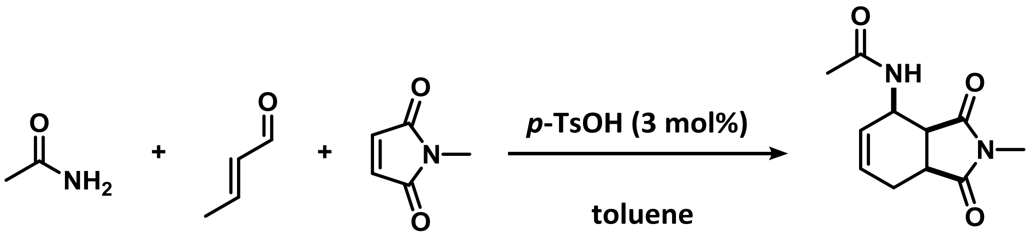

$C_{11}H_{14}N_2O_3$

59.07 **70.09** **111.10** **222.24**

A pressure tube was charged with acetamide (15 mmol, 890 mg, 1.0 equiv), crotonaldehyde (15 mmol, 1.05 g, 1.0 equiv), *N*-methylmaleimide (15 mmol, 1.67 g, 1.0 equiv), *p*-toluene sulfonic acid monohydrate (3 mol %), and toluene (35 mL). The tube was sealed and the reaction was stirred at 110 °C. After 24 h, the solvent was removed under reduced pressure. The crude product was purified flash column chromatography using PE/EA (1:4 to 0:1) to yield **1b** (1.8 g, 54%) as slightly yellow crystals.

TLC: R_f = 0.15 (PE/EA = 1/4)

^{1}H-NMR: (300 MHz, $CDCl_3$): δ [ppm] = 7.29 (d, *J* = 8.4 Hz, 1H), 5.86 (ddt, *J* = 10.2, 7.2, 3.1 Hz, 1H), 5.72 (dt, *J* = 9.5, 3.1 Hz, 1H), 4.79 – 4.58 (m, 1H), 3.34 – 3.07 (m, 2H), 2.94 (s, 3H), 2.71 (ddt, *J* = 15.4, 7.2, 0.9 Hz, 1H), 2.37 – 2.13 (m, 1H), 2.08 (s, 3H).

GC-MS: (m/z): 180 [$MH-C_2H_3O$]$^+$

Batch synthesis of 5-hydroxy-2-methyl-3a,4,5,7a-tetrahydro-1*H*-isoindole-1,3(2*H*)-dione (**3a**)

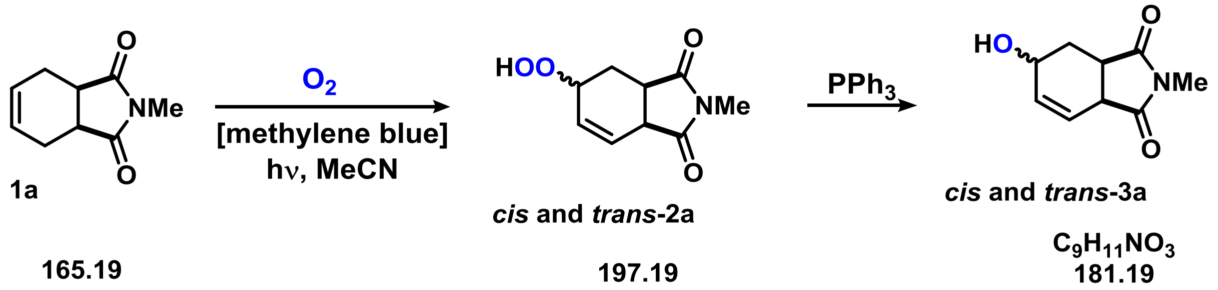

1a (165.2 mg, 1.0 mmol, 1.0 equiv) was diluted in 10 mL of a solution of methylene blue (2×10^{-4} M) in acetonitrile. Pure oxygen was bubbled through the solution while irradiating with white LEDs (6 × Cree MK-R, warm white, 700 mA) for 8 h. The reaction mixture was transferred to a round-bottom flask. Triphenylphosphine (327.5 mg, 1.25 mmol, 1.25 equiv) was added and the solution was stirred for 2 h at rt. The solvent was removed under reduced pressure, followed by purification by column chromatography in PE/EA (2:1 to 1:2) to yield **3a** as pale yellow amorphous solid (148 mg, 82%).

Yield of 3a: 82 % (*trans*/*cis* 5/1)

TLC: R_f = 0.31 (PE/EA = 1/2)

^{1}H-NMR: (300 MHz, $CDCl_3$) δ [ppm] = 6.12 (ddd, *J* = 9.9, 4.0, 2.2 Hz, 0.13H), 6.07 – 5.95 (m, 1.12H), 5.89 (ddd, *J* = 10.1, 4.2, 1.8 Hz, 1H), 4.37 - 4.31 (m, 0.12H), 4.22 – 4.07 (m, 1H), 3.53 – 3.40 (m, 1.12H), 3.21 (dt, *J* = 8.0, 5.7 Hz, 1H), 3.12 – 3.00 (m, 0.14H), 2.98 (s, 0.33H), 2.96 (s, 3H), 2.44 (dt, *J* = 13.1, 4.9 Hz, 1H), 1.76 (ddd, *J* = 13.1, 9.0, 6.1 Hz, 1.18H).

^{13}C-NMR: (101 MHz, $CDCl_3$) δ [ppm] = 178.7, 176.6, 134.9, 122.8, 62.5, 40.9, 36.8, 29.9, 25.0.

FT-IR: $\bar{\nu}$ [cm^{-1}] = 3429, 2945, 1766, 1670, 1435, 1383, 1338, 1282, 1129, 1062, 1006, 828, 716

HRMS: (EI, 70 eV): $[MH]^+$ = 182.0810 (calc. 182.0817)

Compounds **2a** and **3a** were prepared by Tan et al. with similar yields.[3]

Batch synthesis of *N*-(5-hydroxy-2-methyl-1,3-dioxo-2,3,3a,4,5,7a-hexahydro-1*H*-isoindol-4-yl)acetamide (**3b**)

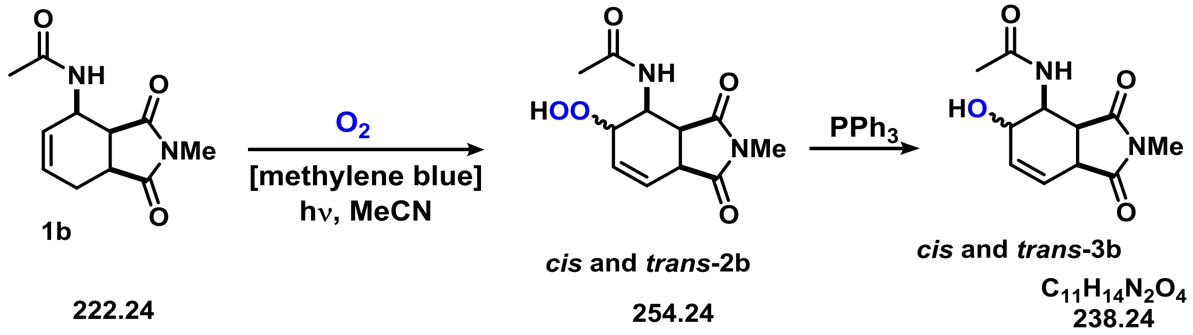

1b (222.2 mg, 1.0 mmol, 1.0 equiv) was diluted in 10 mL of a solution of Methylene blue (2 × 10^{-4} M) in acetonitrile. Pure oxygen was bubbled through the solution while irradiating with white LEDs (6 × Cree MK-R, warm white, 700 mA) for 48 h. The reaction mixture was transferred to a round bottom flask. Triphenylphosphine (327.5 mg, 1.25 mmol, 1.25 equiv) was added and the solution was stirred for 2 h at rt. The solvent was removed under reduced pressure, followed by purification by column chromatography in DCM/MeOH (99:1 to 97:3) to yield **3b** as pale yellow solid (179 mg, 75%).

**Yield:**	75% (*trans*/*cis* >25/1)
**Condition:**	pale yellow solid
**m.p.**	168 °C
**TLC:**	R_f (EA) = 0.14
**^{1}H-NMR:**	(400 MHz, DMSO) δ [ppm] = 7.61 (d, *J* = 8.8 Hz, 1H), 5.92 – 5.83 (m, 1H), 5.83 – 5.70 (m, 1H), 5.20 (s, 1H), 4.15 – 4.01 (m, 1H), 3.92 – 3.72 (m, 1H), 3.62 – 3.52 (m, 1H), 3.45 (dd, *J* = 8.1, 5.9 Hz, 1H), 2.77 (s, 3H), 1.82 (s, 3H).
**^{13}C-NMR:**	(101 MHz, DMSO) δ [ppm] = 177.0, 176.2, 169.1, 132.5, 123.3, 64.1, 49.1, 41.5, 24.3, 22.6.
**HRMS:**	(EI, 70 eV): $[MH]^+$ = 239.1025 (calc. 239.1026)

To the best of our knowledge, **2b** and **3b** are new compounds without any synthetic precedents.

Flow reactions

The flow syntheses of the respective materials were conducted with the explained microreactor setup, applying the conditions mentioned in the text.

References

[1] L. M. Rice, E. E. Reid, C. H. Grogan, *J. Org. Chem.* **1954**, *19*, 884–893.
[2] D. Strübing, H. Neumann, A. Jacobi von Wangelin, S. Klaus, S. Hübner, M. Beller, *Tetrahedron* **2006**, *62*, 10962–10967.
[3] A. Tan, E. Bozkurt, N. Kishali, Y. Kara, *Helv. Chim. Acta* **2014**, *97*, 1107–1114.
